# A Tricky Trait: Applying the Fruits of the “Function Debate” in the Philosophy of Biology to the “Venom Debate” in the Science of Toxinology

**DOI:** 10.3390/toxins8090263

**Published:** 2016-09-07

**Authors:** Timothy N. W. Jackson, Bryan G. Fry

**Affiliations:** 1 Venom Evolution Lab, School of Bioloigical Sciences, University of Queensland, St Lucia QLD, 4072 Brisbane, Australia; tnwjackson@gmail.com; 2 Institute for Molecular Bioscience, University of Queensland, St Lucia QLD, 4072 Brisbane, Australia

**Keywords:** function, venom, evolution, debate, philosophy, science

## Abstract

The “function debate” in the philosophy of biology and the “venom debate” in the science of toxinology are conceptually related. Venom systems are complex multifunctional traits that have evolved independently numerous times throughout the animal kingdom. No single concept of function, amongst those popularly defended, appears adequate to describe these systems in all their evolutionary contexts and extant variations. As such, a pluralistic view of function, previously defended by some philosophers of biology, is most appropriate. Venom systems, like many other functional traits, exist in nature as points on a continuum and the boundaries between “venomous” and “non-venomous” species may not always be clearly defined. This paper includes a brief overview of the concept of function, followed by in-depth discussion of its application to venom systems. A sound understanding of function may aid in moving the venom debate forward. Similarly, consideration of a complex functional trait such as venom may be of interest to philosophers of biology.

## 1. Introduction

The concept of *biological function* has been the source of considerable debate within the philosophy of biology. Similarly, in recent years the concept of *venom*, and the attribution of “venomousness”, has been the source of considerable debate within the science of toxinology. Since venom is generally agreed to be a functional trait, these two debates are conceptually related. An understanding of the debate in the philosophy of biology may help toxinologists clarify their thinking on the subject of venom. Conversely, an understanding of the debate in toxinology may be of interest to philosophers of biology as an example of the difficulties that may arise when attempting to define a diverse functional trait such as venom. Overall, the venom debate is an example of a controversy in science that would benefit from a more active exchange of ideas between science and philosophy of science. This paper begins with a brief overview of the concept of function, as elucidated by philosophers of biology, for the benefit of toxinologists (Table 1). This is followed by an in-depth discussion of the attempt to apply this concept to venom.

### 1.1. Function, “Why” Questions and Teleonomic Explanations

Function is one of the most important concepts in biology because it (along with the related phenomenon of “multiple realisability”) helps to distinguish that science from those it might otherwise be reducible to—chemistry and physics [1,2]. In physics, it is meaningless to ask what things are *for*, they simply *are*—in physics there are only properties, not functions in the sense that they exist in biology. In this sense, as Daniel Dennett [3] has pointed out, biology is more similar to engineering than it is to physics. In biology, the theory of natural selection justifies the asking of *why* questions and permits the use of teleonomic explanations—biologists may ask what a trait’s *purpose* is, i.e., whether it contributes to the achievement of some specific or general *goal* of the organism that possesses it. Specific goals may be the acquisition of food or the avoidance of predators, whereas the general goal of all organisms is understood to be maximising their inclusive fitness. Teleological explanations have long been controversial in the sciences [4]. Function, as a central concept invoked to justify such explanations in biology, has therefore been debated extensively.

Before reviewing the function debate, it is important to clarify what is meant by the reference to why questions and teleonomic explanations in the preceding paragraph, in order to demonstrate that the concept of function is at the very core of these issues. It is possible to provide *scientific* (as opposed to metaphysical etc.) why questions into two major categories, which Dennett has referred to as “how come?” and “what for?” “How come” questions are questions about mechanism, and thus may be referred to as *mechanistic* why questions. This category of why question is legitimately asked in physics, e.g., “*why* does my pen fall to the ground when I knock it off my desk?” No “purposiveness” is sought in the asking of such questions (unless of course you’re doing it on purpose to procrastinate!) and they thus avoid the risk of invoking teleological explanations. “What for” questions, on the other hand, are *explicitly* questions about purpose, e.g., “what does the heart pump blood for?” These are questions about function and may therefore be referred to as *functional* why questions, e.g., “*why* does the heart pump blood?” Or, indeed, “why does the heart *exist*?” These questions may be asked legitimately within biology and it is natural selection that justifies their *telos* (end, goal, or purpose). Teleological (directed towards a goal by *knowledge*) explanations have long been considered taboo in science as they imply the existence of a (Divine) master plan. For this reason, the term teleonomic (goal-directed by *law*) is preferred by some authors [5,6]. Although natural selection is not generally considered a law, it is a process which drives the evolution of purposiveness. The *functional* why question is thus the question that evolutionary biologists attempt to answer in order to explain the existence of specific traits, such as venom.

### 1.2. Theories of Function

Broadly speaking, theories of function either focus on the *causal role* of the trait, or its *evolutionary history* [7] (Table 1). Causal role theories state that the function of a trait is simply what it does (i.e., what it *causes* to happen). This is distinguished from merely its *properties* by the fact that its causal role is said to contribute to, facilitate, or be integral to some higher goal of the system (the organism) of which it is an attribute. Thus, the causal role of a trait might be its contribution to the survival or the fitness (see below) of the organism that possesses it. There are at least two possible difficulties with the causal role account. The first is that it does not explain the presence of a trait—it is silent on how the system came to exhibit the trait in question. Cummins, one of the most influential proponents of this theory considered this to be its particular virtue [8]—it avoids the necessity for teleological explanations of function. According to causal role theory then, functions simply *are*—what they do *now* is what matters, not what they *might* have done in the past. For evolutionary biologists, however, who are precisely concerned with how an organism came to possess a particular trait, this seems to be a considerable weakness. The second limitation of causal role theories is that they are seemingly unable to clearly distinguish between *adaptations* and *epiphenomena* (their distinction between these phenomena is merely pragmatic—[9]). Not all an organism’s traits are necessarily adaptations that have been selected for, some are epiphenomena—“accidental” by-products of selection for other traits, or limitations imposed by the contingency of evolution [10]. For evolutionary biologists, a primary purpose of any theory of function must be to account for the difference between adaptations and epiphenomena, and the Cummins [8] interpretation of the causal role account apparently fails to do this decisively [7]. In evolutionary biology, the teleonomic element of natural selection is of paramount importance.

**Figure 1:**
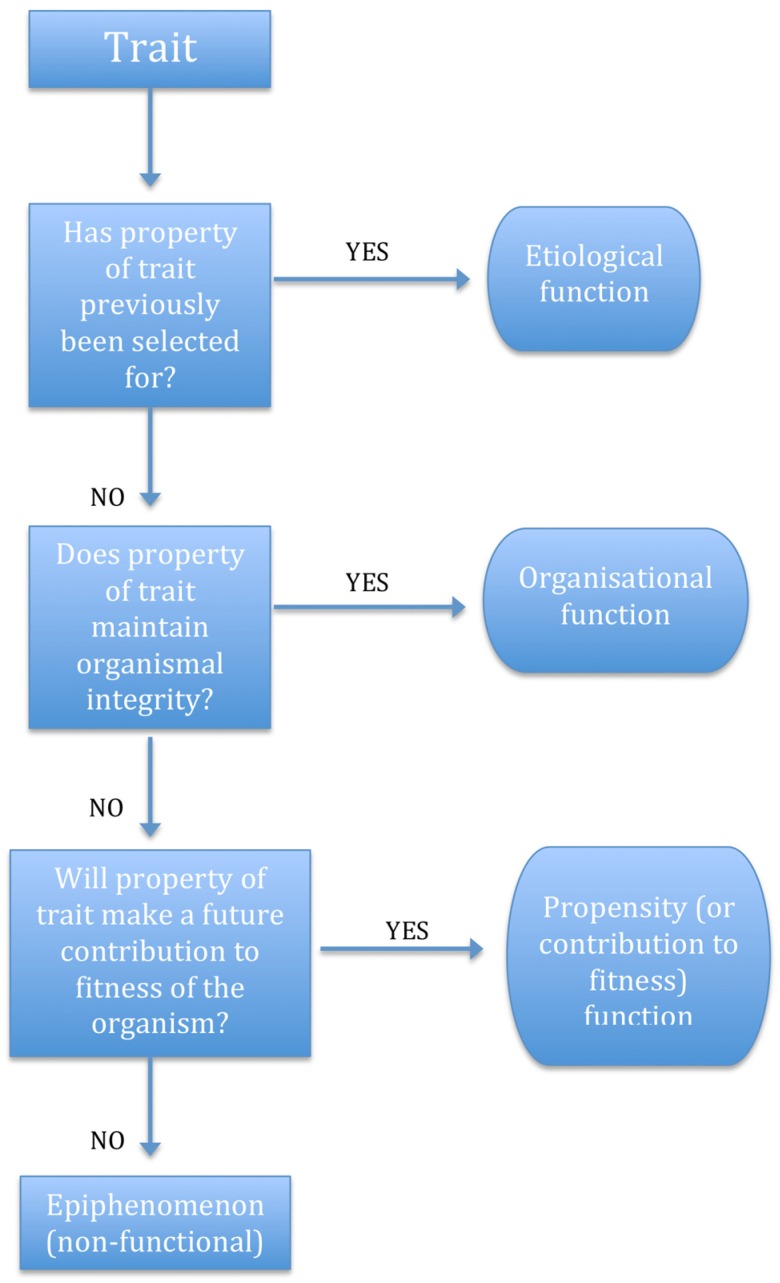
Flow chart illustrating basic application of *evolutionary* theories of function to properties of traits.

Evolutionary theories of function (Figure 1) are concerned with the question of how traits arise and are maintained. Two main categories of evolutionary theories of function are etiological and propensity theories. Etiological theories define the function of a trait according to the past effects of the trait that led to its current presence, i.e., what effect of this trait was selected for over the course of its evolutionary history? Etiological theories seem to do more of the work required by evolutionary biologists than causal role theories, since they attempt to explain the presence of a trait. On the other hand, they are unable to account for the de novo appearance of functions. Essentially, if a trait acquires an additional effect, even if it makes an immediate contribution to the fitness of the organism, an etiological theory would not consider this a “true” function. Although this would be a brief setback, presumably disappearing as soon as the trait was maintained (by selection) into the next generation [11], it represents a weakness of straightforward etiological theories. This weakness is often highlighted by critics that draw attention to the concept of *exaptation* [7]—a trait may be selected for a particular function that inadvertently increases its likelihood of acquiring an *additional* function [12]. These additional functions are not accounted for by etiological theories until/unless they are directly selected for.

Propensity theories, on the other hand, explain functions by appealing to the contribution they make to the *future* fitness of the organism, and are thus able to deal with the appearance of novel functions. In order to do this, however, they appear to sacrifice their ability to explain the trait’s presence, which, as already stated, is of particular interest to evolutionary biologists. Critics of the etiological and propensity approaches to function claim that neither addresses the *current disposition* of a trait, only its past or future, respectively, and that both are therefore powerless to distinguish true functions from epiphenomena [7]. Another view, however, might be that the “causal role” of a trait in its current disposition is the contribution it makes to the future fitness of the organism that possesses it. This apparently pluralistic interpretation would collapse the boundary between causal role and propensity theories.

In order to attempt to deal with this apparent shortcoming of evolutionary theories of function, some theorists favour an *organisational* approach. In this account, a trait’s function is the contribution it makes to “maintaining the integrity” of the system of which it is a part. Organisms are considered to be “self-maintaining” systems and the functions of their traits consist in their contributions to this process of self-maintenance [13]. Organisational accounts have described a function as “a necessary condition” for the existence (self-maintenance) of the function bearer [7]. This seems too strong, however, as organisms are often able to maintain their existence when one or more functional traits are disabled. There is, however, likely to be an impact on the organism’s fitness in such cases, and thus a more generalised *contribution to fitness* approach may capture much of what is useful in the organisational approach [9]. Furthermore, the organisational approach appears to come apart in scenarios in which an organism compromises its maintenance in order to maximise its fitness. There are many such examples in nature, from female octopuses starving themselves to death whilst protecting their brood, to worker bees sacrificing themselves in defence of the hive, to male *Antechinus* that build up lethal levels of testosterone during their quest to maximise breeding success.

Which theory of function is preferable may depend on the context in which it is applied [14,15]. Table 1 provides a basic summary of theories of function for toxinologists to review and Figure 1 illustrates ways in which different evolutionary theories of function might be utilised in order to determine whether or not a specific property of a trait should be considered its function. For evolutionary biologists, an appropriate theory *must* address the functional why question. The answer to questions such as these is *necessarily* teleonomic and appeals *both* to the selective history of the trait (as in etiological theories) and its current disposition in terms of its contribution to the fitness of the organism. The contribution to fitness approach may capture what is useful about both propensity theories (since contributions to fitness will, by definition, be selected for in future) and the organisational approach.

### 1.3. Venom Is a Complex Functional Trait

What is “venom” and which organisms are “venomous”? The property of being toxic is not enough to warrant the classification of a secretion as venom [16]. Although the definition of “venom” has changed considerably over time, the current consensus is that venom is a functional trait and that venomous organisms are defined by their possession of this trait [17]. To make this clear, compare two recent definitions (both from 2012):

“A complex substance produced in a specialized gland and delivered by an associated specialized apparatus that is deleterious to other organisms in a given dosage and is actively used in the subjugation and/or digestion of prey and/or in defense” [18] and “a secretion, produced in a specialised tissue (generally encapsulated in a gland) in one animal and delivered to a target animal through the infliction of a wound (regardless of how tiny it is). A venom must further contain molecules that disrupt normal physiological or biochemical processes *so as to* (emphasis added) facilitate feeding or defence by/of the producing animal” [19].

Although Weinstein [20] states that the latter definition “arguably injects some ambiguity”, this claim seems to stem from a failure to notice the phrase “so as to”, or to recognise that this is a standard way to denote function. Compare “the heart exists *so as to* pump blood” [2]. The two definitions of venom quoted above are equivalent. Thus, the continuing debate about the attribution of “venomousness” to certain organisms does not hinge on the definition of “venom”. Rather, it hinges on the *application* of the definition—i.e., on differences in the understanding and application of the concept of function among toxinologists. In essence, therefore, this is a philosophical debate—can the concepts of function provided to us by philosophy of biology resolve it, or does invoking them risk muddying the waters even further?

## 2. Theories of Function Applied to Venom

### 2.1. The Venom System

**Figure 2:**
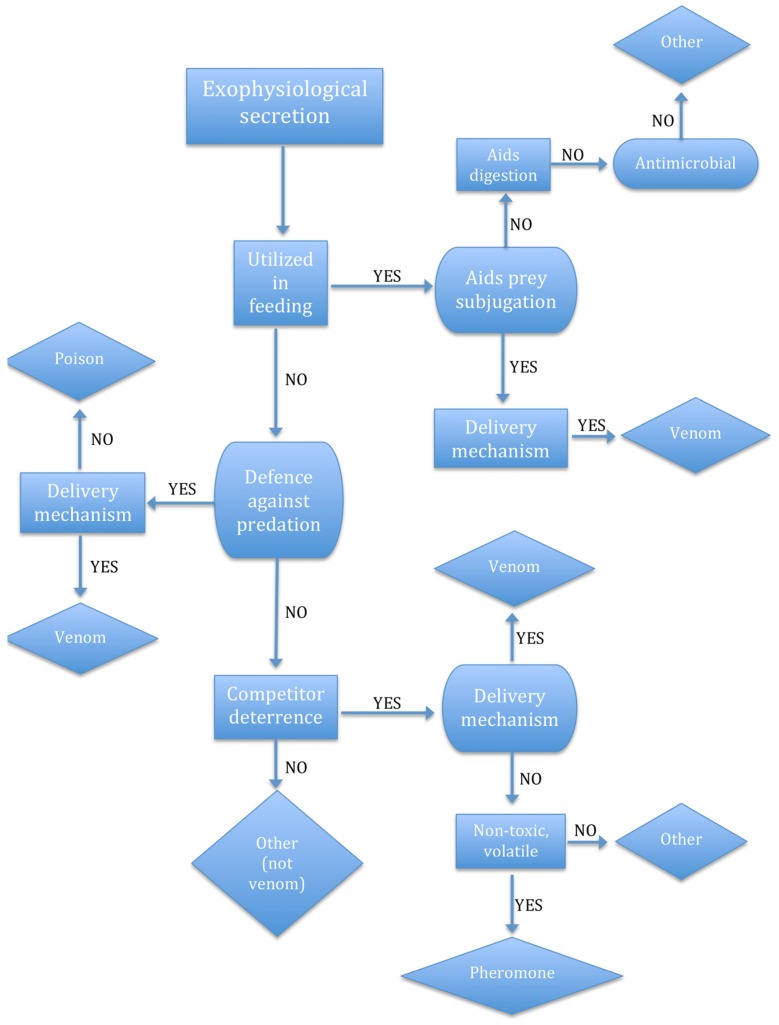
Flow chart illustrating functional analysis of *exophysiological* secretion in order to determine its classification as venom, poison, digestive aid, antimicrobial, or other. An exophysiological secretion is a secretion containing molecules that have evolved to be physiologically active outside the body of the producing organism (e.g., in the body of another organism). Note that this functional analysis is intended as an example only and is not exhaustive.

Applying the causal role theory of function to a secretion would suggest that whatever the effect of that secretion, if it contributed to the furthering of some specific goal of the organism, that effect would be its function. Venom is a secretion that is considered to be an adaptation for feeding, defence, or competitor deterrence. At first glance then, the causal role theory seems suitable for describing the function—if the secretion furthers the goal of feeding, defence, or competitor deterrence, then it is venom. But it’s not this simple, unfortunately. Secretory enzymes may further the goal of feeding by aiding digestion, but these are not venoms. Toxic skin secretions may deter predators, but these are poisons, not venoms. Pheromones may deter competitors, but these are not venoms either. In order to qualify as venom (Figure 2), a secretion must be associated with a *venom delivery system*—a specialised mechanism, the function of which is to deliver venom (note that venom is a specialised form of poison, i.e., poison that is *actively delivered*). This may seem circular—it is difficult to define venom without defining the venom delivery system and impossible to define the venom delivery system without defining venom. It makes more sense, therefore, to consider the “venom system” as a whole, and not try to define each component separately.

### 2.2. Death as an Epiphenomenal Consequence of Envenomation

As previously mentioned, a shortcoming of the causal role account is its inability to differentiate between the function of a trait and its mere *effects*. Since evolutionary history and contribution to fitness are specifically eschewed in many expositions of this account [7,8] it seems difficult to objectively identify the goals that a trait might contribute to. It seems that goals, and therefore functions, are likely to be “observer relative” in this account—the product of the observer’s own perception and not necessarily relevant to the organism.

For example, potential predators or prey, bitten or stung by a venomous organism in defence or offence, may die as a result of the envenomation. Is killing potential predators and prey thus the function of the venom system in this context? In the case of predation, it is hard to see how killing prey makes the goal of consuming it more readily achievable than simply immobilising it would. Indeed, some venoms immobilise prey animals far more rapidly than they kill them, and venomous predators may begin to consume their catch before it dies (e.g., cone snails—[21]). In the case of predator deterrence, is simply causing an unpleasant experience for a would-be predator of greater benefit to a venomous organism (or rather to a venomous species) than killing it? After all, if the predator does not survive its encounter no “learned avoidance” can occur. It seems impossible to gain insight into questions such as these using a causal role theory, which in both cases would have us conclude that killing is indeed (one of) the venom system’s function(s).

In the case of predation, selection pressures cannot “see” the difference between a dead prey item and one that is “merely” completely subdued. Selection simply works on the efficacy of the venom system in aiding the predator’s attempt to secure a meal. Thus, killing an intended prey item prior to its ingestion makes no contribution to the fitness of the predator. According to an evolutionary account of function, for predatory venoms at least, killing is an *epiphenomenon*—an unselected by-product of the fact that most pharmacological means of completely subduing a prey animal also result in its death (not all do, e.g., parasitic wasps—[22]).

In the case of defensive applications of venom systems, insistence that learned avoidance is crucial would ignore the fact that selection may act directly on *replicators* (genes) and only indirectly on their *vehicles* (individual organisms) [23,24]. Many species that use their venom defensively exhibit aposematic colouration [25]. If their venom kills individuals with a genetic predisposition for being incautious and ignoring warning signals, then genes for cautiousness will be positively selected and the population of predators will come to be dominated by cautious individuals. Thus venom that kills may indeed make a contribution to the (inclusive) fitness of organisms that use it in defence—the evolutionary account of function agrees with the causal role account in this case. On the other hand, many venomous creatures that use their venom solely for defence have venoms that appear designed to cause pain and inflammation, but certainly not to kill [26]. So, learned avoidance may be important in the evolution of defensive venoms after all. When venom is used both in defence and in prey capture, however, there is no salient reason why killing potential predators would be selected against. Indeed, avoidance of aposematic prey may be learned *or* genetically acquired [27], or perhaps both in some cases.

### 2.3. Use It (for Predation) or Lose It

The fact that venom may often have multiple functions is another reason that it can be a tricky trait for functional analysis. Taking again the example of a venom system that is used for both predation and defence, as appears common to many snake species, we can see that different theories of function might yield different answers concerning its functionality. A causal role theory will of course determine that both predation and defence are functions of the venom, but once again this approach may conceal much of what is of interest to evolutionary biologists. At first glance it might appear uncontroversial that both putative functions would make a contribution to the fitness of the snake, but real-world evidence appears to contradict this. In actual fact, snakes that do not use their venom for predation seem to undergo a reduction of the venom system [19]. The most clear-cut examples of this “use it (for predation) or lose it” phenomenon are the specialist egg-eating sea snakes (*Aipysurus eydouxii, Aipysurus mosaicus* and the three species of *Emydocephalus*) [28,29]. Although descended from elapid snakes with exceptionally advanced venom systems, these snakes have (independently in each genus) all but lost their venom glands and fangs, apparently following the transition to a “defenceless” diet. The sequences of toxins still expressed by their venom glands have also rapidly accumulated deleterious mutations, greatly reducing their toxicity [30]. This suggests that, for sea snakes at least, the defensive role of the venom system does not make a contribution to the fitness of the snake sufficient to warrant its maintenance after the predatory role is lost. Sea snakes will certainly use their venom systems defensively (e.g., biting fishermen when caught in trawl nets—[31]), but does this warrant considering defence a function of their venom? Utilising an evolutionary account of function may convince us otherwise—it is merely an epiphenomenon.

The use it or lose it evolutionary trajectory of snake venom systems is not limited to sea snakes—it appears to be a common phenomenon. Many species of snake that feed on defenceless prey or switch to constriction as a primary (as opposed to utilising constriction in concert with venom) means of subduing prey have considerably reduced venom glands in comparison to close relatives [19]. As well as indicating that a defensive role for venom is epiphenomenal in some snakes, this constitutes compelling evidence for the fact that venom systems have a predatory function for a diverse range of non-front-fanged snakes with well-developed venom glands. Perhaps, however, decreeing that the defensive role of venom systems is epiphenomenal for many snakes is premature. Even if this role does not make a *sufficient* contribution to justify the presumed metabolic cost of the venom system in the absence of a predatory role, it may still make a small contribution to fitness whilst it is being maintained for predation.

### 2.4. The Context-Specificity of Functions

Another difficulty with the classification of functional traits is the fact that their effects may be *context-specific* [14]. This is commonly the case with venoms and their constituent toxins, which may be highly specialised for targeting specific prey types, i.e., they may be exceptionally toxic to some species and only mildly or non-toxic to others (e.g., irditoxin from *Boiga irregularis*—[32]).

Consider the case of a snake that specialises in feeding upon lizards, subduing them rapidly with highly toxic venom. If this snake somehow becomes isolated on an island with only mammalian prey, against which its venom is ineffective, and has to resort to constriction to secure a meal, is it still a “venomous” snake? A causal role or contribution to fitness theory of function might suggest that it isn’t, despite the fact that it possesses a sophisticated venom delivery system that evolved in order to deliver the toxic oral secretion it so recently used to subdue its prey. An etiological theory would deliver the opposite verdict. Imagine that instead of an individual, a small population of these snakes becomes isolated on this lizardless island. All of them are initially forced to resort to constriction, but, being relatively gracile snakes, they aren’t very good at it—they can only secure small meals and often fail to subdue their prey at all. In the secretion of their (erstwhile venom) dental glands, along with a cocktail of toxins that rapidly immobilises lizards, but has no effect on mammals, is an enzyme with antibacterial activity. Typically this enzyme is secreted in very low quantities, because not much is needed to keep the snake’s mouths free of pathogens. In a small number of individuals, however, for purely contingent reasons, this enzyme is secreted more abundantly. When these lucky individuals bite a mammal and begin to constrict, the antibacterial enzyme has a previously unselected side effect—it weakens mammals, making them easier to subdue. Is subduing mammals now a *function* of this enzyme? An etiological theory would supply a negative answer to this question; casual role or propensity theories a positive answer. These snakes secure larger and more frequent meals than their counterparts and thus will outcompete them. The enzyme’s *exaptation*, therefore, makes an immediate *contribution to their fitness*—ultimately the genes responsible for higher expression levels of the enzyme will go to fixation and the island population as a whole, once again “venomous” by anyone’s standards, will be characterised by a different venom composition than that of the mainland population.

The above story may be read as a subtly cautionary tale for those who wish to determine the “venomousness” of snakes by utilising prey-handling experiments in the laboratory—if these are not conducted using natural prey items the conclusions that may be justifiably drawn from them are limited (cf. [33]). Such experiments can make a valuable contribution to our knowledge of snake prey-handling behaviour, but close attention must be paid to the context in which this behaviour evolved.

### 2.5. Defective Functions—Vestigial and Incipient Traits

The issue arising from the context-specificity of certain functions is an example of the more general problem of *defective* functions—when a trait is unable to perform a role for which it is typically responsible, is this role still its function [34]? This question is highly relevant to evolutionary biologists because of the existence of *vestigial* traits. The “venom glands” of egg-eating sea snakes are again a suitable example. Their considerable reduction in size and the accumulation of deleterious mutations in the toxins they express is evidence that they are no longer under selection pressure to produce venom. They are not, therefore, making a contribution to the fitness of the snakes by producing venom. Nor are they, in fact, exhibiting the causal role of venom production. So, neither a contribution to fitness nor a causal role theory of function should consider producing venom their function. The case is slightly more complicated with etiological and propensity theories. Since the evolutionary history of the glands included selection for venom production, and since that history explains their (albeit reduced) presence, an etiological theory should still consider their function to be venom production. Application of a propensity theory delivers ambiguous results—although it initially seems obvious that they will not make a future contribution to fitness by producing venom, this is not at all clear. It is certainly possible for these snakes to be forced into another shift of dietary preference, one that might see selection of their dental glands for venom production resume. From this perspective utilisation of a propensity theory might see vestigial venom glands retaining “honorary” functional status.

Things are further convoluted when the question of *incipient* traits is raised. Incipient traits are essentially exaptations—traits that, either through adaptation for some other function, or purely epiphenomenally, have properties that predispose their co-option for some additional or alternative function [12]. Incipient traits may thus make a contribution to future fitness via a causal role they do not currently possess. Obviously, different theories of function will analyse them differently, again highlighting the observer relativity of functional attributions. Observer relativity becomes an even greater influence in the determination of whether or not a trait is in fact incipient, vestigial, or neither, perhaps fulfilling some *distinct* functional role that the investigator has yet to uncover. How can we determine if a trait is incipient or vestigial in reference to a specific function of interest (disregarding the possibility of alternative functions for the purposes of analysis)? The dental glands of iguanian lizards have been referred to as “incipient venom glands” [19,35], whilst the homologous glands of egg-eating sea snakes are considered vestigial. Both sets of glands express secretory proteins homologous with characterised venom toxins, although neither group appears to utilise venom for prey capture or defence. It is only knowledge of organismal phylogeny, and the application of the parsimony principle, that can aid us to make judgement that the former are incipient and the latter vestigial.

### 2.6. Function Categories

Organs are often named according to their membership of *function categories* [34]—“hearts” are *for* pumping blood, “eyes” are *for* seeing, “wings” are *for* flying, and so on. Note that the members of a function category are not necessarily developmentally or structurally homologous—the vertebrate heart is not homologous with the branchial heart of coleoid cephalopods; the vertebrate eye is not homologous with the compound eye of insects; bird wings are not homologous with insect wings, etc. It is solely the recognised *function* of the organ that places it within its category. Similarly, “venom glands” are *for* producing venom, and those of reptiles are not homologous with those of insects, arachnids, chilopods, fish, etc.—venom glands have evolved many times independently throughout the animal kingdom [26]. Even when members of a category *are* developmentally homologous, there may be considerable structural variation between them. Reptilian hearts (except for those of crocodylians) have only three chambers, unlike the homologous mammalian (and crocodylian) hearts, which have four.

Similarly, there is a great deal of well-documented variation in the structure of reptilian venom glands, including those of the three clades of front fanged snakes (Atractaspinae (including *Homoroselaps*), Elapidae, and Viperidae). This variation exists both between and within these clades, and is independent of the variation of other components of the venom delivery system, such as the development of the fangs. For example, the venom glands of the front-fanged elapid snakes are structurally more similar to those of some non-front-fanged “colubrid” snakes (members of the polyphyletic assemblage formally classified as the Colubridae) [19,36]. Similarly, the venom glands (and fangs) of *Homoroselaps* are so similar to those of the Elapidae that this genus (now known to be more closely related to *Atractaspis* [37], the venom system of which is quite different) was formally classified as part of the Elapidae [38,39]. Although some authors have suggested that front-fanged snakes are the only reptiles with “true” venom glands (despite agreeing that certain non-front-fanged snakes are “venomous”—[17]), we believe that reptile venom glands should constitute a normal functional category and that glands that produce venom are, *ipso facto*, “venom glands”.

### 2.7. Vestigial and Incipient Traits Revisited

An obvious consequence of the difficulty incipient and vestigial structures present for functional analysis is the fact that assigning them to functional categories may be controversial. A similar difficulty is encountered with *borderline* cases [34]. Vestigial structures are often relatively easily dealt with (assuming their “vestigiality” is itself uncontroversial)—naming the vestigial and non-functional (for flight) wings of ratite birds provokes few arguments. Similarly, referring to the dental glands of egg-eating sea snakes as “venom glands” is unlikely to raise many eyebrows. Incipient organs present a more obvious problem because they draw more attention to the observer relativity of functions. Are the ocelli of flatworms “incipient eyes”? What about the parietal eyes of lizards? Is any conglomeration of photoreceptors an incipient eye? Clearly not, but where do the incipient eyes “begin”? The problem with classifying organs as incipient in relation to a specific function is that they may be fulfilling some other functional role not being considered by the investigator. It has been claimed that this “may be diverting attention from (these) other possible roles” [18].

In fact, the observer relativity of functional *labelling* is ever present, and is certainly equally problematic when classifying organs as vestigial. Is the human appendix vestigial, or is it a haven for the proliferation of beneficial gut flora [40]? If an ostrich uses its vestigial wings to help maintain its balance while running at top speed or in a threat display to potential predators, or in courting potential mates, is it “fair” to refer to them as “vestigial wings” at all? The incipient venom glands of iguanian lizards may produce antibacterial proteins or help condition the lizard’s teeth [35]. Might not calling them “incipient venom glands” obscure these possible functional roles?

These apparent problems should disappear when it is acknowledged that these labels may be considered *stipulative.* Stipulative definitions those utilised for the purposes of a specific argument or hypothesis. They are contrasted with *descriptive* and *theoretical* definitions [34]. Descriptive definitions describe the specific attributes of a phenomenon (in this case a functional trait) that are important in defining it—the definitions of venom quoted earlier in this paper are descriptive definitions. Theoretical definitions, on the other hand, are empirically validated *explanations* of a phenomenon, usually in terms of reduction, e.g., “water is composed of two hydrogen atoms and an oxygen atom”; “malaria is caused by protozoans of the genus *Plasmodium,* most commonly vectored by female *Anopheles* mosquitoes”. Acknowledging that a label is stipulative does not undermine its usefulness, but draws attention to the importance of the context in which it is used. “Incipient” and “vestigial” are terms that refer only to trait’s properties in relation to some specific “function of interest” and certainly do not mean “devoid of additional function”. Many traits and organs possess multiple functions. Birds that retain the power of flight may also use their wings for displaying to mates (e.g., riflebirds) or potential predators (e.g., bush stone curlews, cranes etc.). These are classic examples of exaptation, in which the evolution of one functional trait provides the substrate for the evolution of another—wings that evolved for flight may easily become co-opted for use in displays. Similarly, the antibacterial function of the dental glands of an ancestral toxicoferan lizard may have provided the substrate for the evolution of venom [41].

It is necessary to dwell on the subject of incipient traits a little longer in order to clarify two further potential sources of confusion. Firstly, “incipient venom glands” are not necessarily “venom glands”, that is, they do not *necessarily* produce venom. Secondly, incipient venom glands are not necessarily “in the process” of evolving into venom glands. They are given this label to highlight the fact that they are developmentally homologous (with venom glands) secretory glands that are present in a clade of lizards closely related to all those (including snakes) that possess venom glands. They are associated with the dental lamina and express proteins of the same classes as some of those expressed in well-characterised venom glands. They are thus of considerable interest to evolutionary biologists studying the evolution of the venom system of squamate reptiles. Their function remains enigmatic, but their relatedness to venom glands is well established [19,36,42]. It may be controversial to refer to an organism as “venomous” without appropriate evidence of the functional deployment of venom, but referring to their dental glands as “incipient venom glands”, in light of the above discussion, should not be—it is not dissimilar to referring to them as *plesiomorphic* (similar to an inferred ancestral state).

## 3. Conclusions

In science, as in philosophy, debate may often be stimulated more by disagreements regarding the usage of specific words, rather than directly by the data being analysed. In *Phaedrus*, Plato [43] claimed that nature is pre-divided; composed of essential “Forms”, and that the job of theories is to “carve nature at its joints” [44]. In practice, however, it can be extremely difficult to locate these joints, which mark the boundaries between “natural kinds”. Indeed, debate within the philosophy of science about the *existence* of natural kinds and whether our concepts can accurately map them is as lively as the debates about function and venomousness [44,45]. An example of a debate about natural kinds that is familiar to evolutionary biologists is the “species problem” [2,46]. Discrete categories with binary relationships, such as “venomous” versus “non-venomous” are easily generated as labels, but may not be so clearly defined in nature. Appropriate usage of terminology is critically important to the collaborative endeavour that is science, but it is equally important that constructive debates do not devolve into arguments about the placement of boundaries. Such placement may ultimately, either due to epistemic gaps or because the boundaries simply do not exist, be arbitrary.

In order to discuss the application of functional terminology, it is necessary for scientists to have at least rudimentary knowledge of the debate that has taken place on this topic in the philosophy of biology. Similarly, participants in this philosophical debate must also pay due attention to the complexities of functional traits uncovered by empirical investigation. Although and in-depth consideration of the subtleties of the various concepts of biological function is beyond the scope of the present essay, it is likely that no single one amongst those most popularly defended can adequately describe a functional trait such as venom in all its evolutionary contexts and extant variations. For this reason, some philosophers of biology have advocated a pluralistic concept of function, recognising the fact that different interpretations of function may be more or less appropriate to certain contexts [14,15]. For evolutionary biologists, both the past and (potential) future contributions to an organism’s fitness that a trait makes are important in defining that trait’s function(s). The most relevant parts of several different theories may need to be invoked—different concepts may be more effective at describing different instances. When proposing evolutionary hypotheses, the freedom to use stipulative definitions is also important. Ultimately, the usefulness of theories of function, and of labels (stipulative, descriptive or theoretical) such as “venomous”, is in aiding us to further our understanding of the evolutionary history and current disposition of the trait in question, not in allowing us to defend the lines we have drawn in the sand.

The debates in toxinology and philosophy of biology will continue, but each can benefit from paying attention to the other. Like the species problem, both venomousness and function in general are quintessential examples of the difficulties we are likely to encounter when we attempt to carve nature where there may not be any “salient joints” and we resort to “approximating confabulation” [47]. Venom and many other functional traits exist as spectra, with each instance of the trait just a point on the continuum. Creationists may ask, “What is the use of half an eye?” To which we may reply, “Half an eye is very useful indeed!” Similarly, venomous organisms may receive a small and perhaps undetectable (through observation or experiment) contribution to fitness from a toxic secretion that makes prey ever so slightly easier to subdue, or they may be completely reliant on their venom in order to secure a meal. Differentiating between *borderline*, *defective* and simply *non-*functional cases may not always be possible in practice. The most sensible approach, when studying the evolution of a complex trait, seems to us to be to identify the largest observable shifts and label them appropriately. Then, once the start and end points of an evolutionary continuum have been defined, clear evolutionary hypotheses can be devised and tested. In the case of the evolution of the venom system of squamate reptiles, the earliest major shift appears to have occurred in the common ancestor of the Toxicofera. Whether this ancestor was itself “venomous” may not be a question that it is possible to definitively answer, but what seems clear is that it possessed uniquely developed dental glands which are the synapomorphy of its ancestors (the extant Toxicofera) and the substrate for the evolution of all known reptilian venom systems. In order to move forward in our investigation of this remarkable system, we should not attempt to split it arbitrarily into many discrete categories, but should recognise its single origin and that all of its myriad extant forms exist as points on the same continuum.

## Figures and Tables

**Table 1:** Brief description of popular theories of function and their potential limitations.

Theory	Defines “Function” as	Potential Limitations
*Causal role*	Effect of trait that contributes/facilitates/is integral to *achievement of organismal ‘goals’*.	Potential failure to distinguish between *adaptations* and *epiphenomena*.
*Etiological*	Effect of trait selected for during the evolutionary *past* that explains its current presence.	Inability to account for de novo appearance of functions.
*Propensity*	Effect of trait that makes contribution to the *future* fitness of the organism that possesses it.	Failure to consider *current disposition* of trait.
*Organisational*	Effect of trait that contributes to *maintaining the integrity* of the system (organism) that possesses it.	Conflict between *maintenance* of organism and maximisation of organism’s *fitness*.
*Contribution to fitness*	Effect of trait that makes a *contribution to the fitness* of the organism that possesses it.	Must include considerations of the trait’s evolutionary history as well as its current disposition
